# Collective Emotional Contagion in Zebrafish

**DOI:** 10.3389/fnbeh.2021.730372

**Published:** 2021-09-09

**Authors:** Daniel Alberto Burbano Lombana, Simone Macrì, Maurizio Porfiri

**Affiliations:** ^1^Department of Mechanical and Aerospace Engineering, Tandon School of Engineering, New York University, Brooklyn, NY, United States; ^2^Centre for Behavioural Sciences and Mental Health, Istituto Superiore di Sanità, Viale Regina Elena, Rome, Italy; ^3^Department of Biomedical Engineering, Tandon School of Engineering, New York University, Brooklyn, NY, United States; ^4^Center for Urban Science and Progress, New York University, Brooklyn, NY, United States

**Keywords:** behavior, Citalopram, empathy, geotaxis, transfer entropy

## Abstract

Seeking to match our emotional state with one of those around us is known as emotional contagion-a fundamental biological process that underlies social behavior across several species and taxa. While emotional contagion has been traditionally considered to be a prerogative of mammals and birds, recent findings are demonstrating otherwise. Here, we investigate emotional contagion in groups of zebrafish, a freshwater model species which is gaining momentum in preclinical studies. Zebrafish have high genetic homology to humans, and they exhibit a complex behavioral repertoire amenable to study social behavior. To investigate whether individual emotional states can be transmitted to group members, we pharmacologically modulated anxiety-related behaviors of a single fish through Citalopram administration and we assessed whether the altered emotional state spread to a group of four untreated conspecifics. By capitalizing upon our in-house developed tracking algorithm, we successfully preserved the identity of all the subjects and thoroughly described their individual and social behavioral phenotypes. In accordance with our predictions, we observed that Citalopram administration consistently reduced behavioral anxiety of the treated individual, in the form of reduced geotaxis, and that such a behavioral pattern readily generalized to the untreated subjects. A transfer entropy analysis of causal interactions within the group revealed that emotional contagion was directional, whereby the treated individual influenced untreated subjects, but not vice-versa. This study offers additional evidence that emotional contagion is biologically preserved in simpler living organisms amenable to preclinical investigations.

## Introduction

Interacting with individuals displaying certain emotional states can elicit similar states in other group members. This phenomenon is known as emotional contagion and is known to be of paramount importance to understand the underpinnings of cognition, emotion, and behavior (Hatfield et al., 1994; Von Scheve and Salmella, 2014). Indeed, primitive emotional contagion is considered a building block of social interactions through empathy (Decety and Ickes, 2011; Von Scheve and Salmella, 2014). The spread of emotions can be as contagious as the spread of a virus (Von Scheve and Salmella, 2014) and, depending on the nature of the emotion and the social context, emotional contagion can be detrimental or beneficial. For instance, in Barsade (2002), it has been shown that the spreading of positive emotions in human groups improved cooperation and decreased conflict in a working setting, while in Bakker and Schaufeli (2000) it was observed that burnout spreading was more likely if teachers frequently shared with each other work-related problems.

Evidence of emotional contagion has been documented across different species and taxa (Brothers, 1989; Palagi et al., 2020). In non-human animals, emotional contagion has been extensively investigated in mammals and birds. For instance, in early experiments with Rhesus macaques (Mirsky et al., 1958; Miller et al., 1963), it was found that fear induced by a conspecific can be transmitted to observer monkeys in a separate cage. These early findings were mechanistically backed by the discovery of mirror neurons, a set of neurons that fire when a monkey observes an action performed by another individual (Ferrari et al., 2003).

In rodents, there is a vast literature documenting empathy, which under Preston and de Waal’s definition is viewed as a collection of stacking dolls where emotional contagion is at the center and each layer represents a higher level state, ranging from cognitive empathy to prosocial behavior (Mogil, 2012, 2019). Within this context, Langford et al. (2006) studied emotional contagion of pain in mice tested in dyads. They found that familiarity modulates pain transmission and that vision is the salient sensory modality through which this transmission occurs. Similarly, Gonzalez-Liencres et al. (2014) demonstrated that a mouse observing another mouse in distress can experience similar emotions and that this contagion is modulated by familiarity. Zoratto et al. (2018) further extended these observations by proving that emotional contagion in mice is sensitive to the intranasal administration of oxytocin, a hormone that modulates social behavior. Recently, emotional contagion has been also documented in dogs (Palagi et al., 2015) and ravens (Adriaense et al., 2019).

In fish, evidence of emotional contagion is not as vast as in other species (Pérez-Manrique and Gomila, 2021). It has been argued that fish possess limited cognitive capabilities that compromise their ability to experience and react to the distress and suffering exhibited by conspecifics (Rose, 2002; Rose et al., 2014). Nevertheless, recent evidence on the zebrafish animal model showed that emotional contagion may extend to the fish taxon. For instance, Oliveira et al. (2017) observed that a shoal of zebrafish exhibiting antipredatorial responses to the imminent threat associated with visual exposure to a live predator elicited a fear reaction in bystanders. Likewise, Silva et al. (2019) induced a fear reaction in a demonstrator zebrafish through an alarm substance and then measured the behavior of a bystander, swimming in a separate tank. The authors showed that the bystander exhibited prompt fear reaction despite swimming in water devoid of alarm substances and that the underlying emotional contagion was stronger in familiar, rather than unfamiliar pairs.

Zebrafish is a tropical freshwater species that has been utilized as a model organism to answer pressing questions in translational neuroscience (Stewart et al., 2014) and in biomedical research to study human diseases (Choi et al., 2021), among many other disciplines (Norton and Bally-Cuif, 2010; Fontana et al., 2018). A wide range of key advantages, such as easy maintenance and high reproduction rate (Nusslein-Volhard and Dahm, 2002), high homology to humans (Panula et al., 2010), and fully sequenced and annotated genome (Howe et al., 2013), have made zebrafish rise as a species of choice in preclinical research.

Here, we seek to contribute to the study of emotional contagion in zebrafish along two research strands that have received marginal attention within existing literature. First, we focus on the reduction rather than an increase of anxiety-related responses as the target emotion to be propagated in the group. Second, we overcome the demonstrator/bystander dichotomy to explore complex interactions that emerge in a group where the behavior of one individual defers from the norm. To reduce the anxiety-related behaviors of one of the group members, that subject was administered Citalopram, a selective serotonin reuptake inhibitor with known anxiety-reducing effects in zebrafish (Sackerman et al., 2010; Macrì et al., 2019; Clèment et al., 2020). In light of the strong effect of Citalopram on geotaxis, which is a very sensitive measure of anxiety-related state (Kalueff et al., 2013), we devised an experimental setup through which this response was maximized. Owing to our in-house developed multi-target tracking software (Butail et al., 2013; Bartolini et al., 2015), capable of preserving fish identities over time, we obtained swimming trajectories of all subjects and assessed their individual and social behavioral phenotypes. We hypothesized that an individual experiencing lower levels of anxiety due to Citalopram treatment can trigger similar emotions on the group and that this behavior is modulated by the Citalopram concentration.

## Materials and Methods

### Animal Care and Maintenance

A total of 216 wild-type zebrafish (*Danio rerio*), with a heterogeneous genetic background and an average size of 3.5 cm, were used in this study. The fish were purchased from Carolina Biological Supply Co. (Burlington, NC, USA). Following standard recommendations (Avdesh et al., 2012; Aleström et al., 2020), we housed the fish in a 615 L vivarium [180 cm (length) × 60 cm (height) × 87 cm (width)] at the stocking density of ~3 fish/L. The vivarium was equipped with two canister filters; one Fluval FX6 (Fluval, Hagen Inc, Montreal, Quebec, Canada) and another one Penn-Plax Cascade (Penn-Plax, Hauppauge, NY, USA). Water parameters of the holding tank were regularly checked, and temperature and pH were maintained at 27°C and 7.2, respectively. Regular tap water was used with the addition of a stress coat (AquaSafe plus, Tetra, Spectrum Brands Inc., Sulzbach, Germany) to remove chlorine and chloramines. We also added API Quick Start freshwater and saltwater aquarium Nitrifying bacteria (API, Mars Inc., McLean, Virginia, USA) to enhance the initial cycling of the tank. We kept male and female subjects separated using a transparent perforated separator placed in the middle of the tank. Such a separation allowed the identification of male and female subjects to be used in the experiments. This procedure was aimed at maintaining the experimental population balanced across sex in all experimental conditions. Fish were maintained on 12 h light/12 h dark photo-period and they were fed with commercial flake food once a day at 7 PM. Prior to the beginning of the experiments, fish were acclimatized in the holding facility for approximately 1 month.

### Experimental Setup, Pharmacological Treatments, and Experimental Conditions

The experimental set-up consisted of a rectangular tank, a video camera (Logitech C910 HD Pro Webcam, Logitech, Switzerland) located in front of the tank, an array of lights, and black curtains surrounding the test area to minimize potential disturbance from cues outside the experimental tank. An experimental test section of 75 cm × 10 cm × 35 cm (length, width, and height) was arranged inside the tank using corrugated white plastic. The tank width and depth were selected to promote swimming along the water column. Such decision rested upon the hypothesis that SSRIs generally result in an overt alteration of geotaxis (Sackerman et al., 2010; Macrì et al., 2019; Clèment et al., 2020); this strategy has been borrowed from available literature (Cachat et al., 2011; Mathuru et al., 2017).

Experimental subjects were administered a water solution of Citalopram (Citalopram Hydrobromyde, Sigma-Aldrich, Burlington, USA) of 0 mg/L (control), 30 mg/L, and 100 mg/L. Based on available literature (Sackerman et al., 2010; Macrì et al., 2019), we administer Citalopram through immersion of the experimental subject in a 500 ml beaker with the desired concentration for 5 min. Adopting this procedure, Sackerman et al. (2010) observed the presence of a direct correlation between Citalopram concentrations in the beaker and in zebrafish brain following a 3–4 min exposure. Citalopram concentrations were selected based on our previous work (Macrì et al., 2019; Clèment et al., 2020) wherein we observed an alteration in anxiety-related behaviors in response to Citalopram administered at the chosen concentrations.

**Figure 1:**
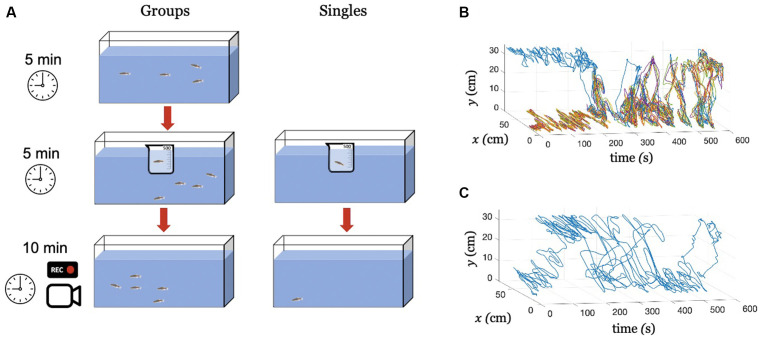
Experimental procedure and tracking. **(A)** An illustration of the experimental procedure for conditions Groups and Singles. Exemplary tracked swimming trajectories over time, **(B)** Condition Groups, where the blue color identifies the treated individual and all the other colors the untreated subjects, and **(C)** Condition Singles, where the blue color identifies the treated fish.

To investigate the extent to which Citalopram-induced behavioral alterations translated to untreated group members, we tested experimental subjects under two main conditions: Singles (subjects tested in isolation), and Groups (a treated subject tested in the presence of four untreated conspecifics). The experiments were performed between November 20, 2020 and December 10, 2020. Overall, our study consisted of two testing conditions (Singles and Groups) and three treatments [0 mg/L (control), 30 mg/L, and 100 mg/L]. A schematic of the experimental procedure is shown in (Figure 1A). Twelve trials were conducted for each treatment/condition yielding a total of 72 trials counterbalanced across sexes (Singles and Groups). Thus, the experimental population consisted of 36 male and 36 female zebrafish. The 72 trials were evenly distributed over 6 days, keeping always the same number of tests per day (six in the morning and six in the afternoon). All fish were experimentally naïve, and were used only once.

For condition Singles, at the beginning of the trial, a single subject was transferred from the housing tank to a 500 mL beaker containing the desired concentration of Citalopram. The beaker was placed inside the experimental tank and the subject was kept there for 5 min following our previous work (Macrì et al., 2019). Then, the subject was transferred to the experimental tank and its behavior was video recorded for 10 min. Similarly, for condition Groups, at the beginning of the trial, four subjects were transferred from the housing tank into the experimental tank and left therein for 5 min. Towards the end of this phase, the treated subject was transferred from the housing tank to a 500 mL beaker containing the desired Citalopram concentration. The beaker was immediately placed inside the experimental tank and the subject was kept there for additional 5 min. Finally, the subject was transferred to the experimental tank and the group behavior was video recorded for 10 min.

### Video Tracking

All videos were recorded at 30 frames per second yielding a total of *N_s_* = 18,000 images of 1,280 × 720 pixels. Images were input to a Matlab-based multi-target tracking algorithm Peregrine (Butail et al., 2013; Bartolini et al., 2015). The software fitted a parabola on the fish blob and returned time series of fish centroid coordinates ${\left\{x(t)\right\}}_{t=1}^{N_{s}}and{\left\{y(t)\right\}}_{t=1}^{N_{s}},$ along with their velocity components ${\left\{v_{x}(t)\right\}}_{t=1}^{N_{s}}and{\left\{v_{y}(t)\right\}}_{t=1}^{N_{s}},$ with *t* representing the time step. Here, *x*(*t*) and *y*(*t*) correspond to the position across the horizontal plane and along the water column, respectively. Each time series consists of *N_s_* samples corresponding to the total experimental time of 600 s (10 min). Exemplary tracked trajectories for conditions Groups and Singles are shown in Figures 1B,C, respectively.

### Behavioral Scoring

The output of the tracking system was used to investigate fish behavior through the scoring of salient metrics. In particular, geotaxis was quantified by computing the time average of the distance from the bottom of the tank. Group cohesion and coordination were assessed through the average nearest neighbor distance and alignment of the swimming direction (Theodorakis, 1989; Parrish et al., 2002; Miller and Gerlai, 2012). In particular, we computed the average nearest neighbor distance between the treated subject and the untreated ones, and between the untreated subjects only (excluding the treated individual; Rosa et al., 2020). The former was computed as the average of the minimum distance between the treated subject and each of the four untreated fish over the total experimental time of 10 min. The latter was computed as the average of the minimum distance between the four untreated subjects over the total experimental time of 10 min. To quantify the alignment between the four untreated subjects, we computed the time average of the instantaneous polarization given by Aureli et al. (2012)

$$Pol(t)=\frac{1}{N-1}\left\|\sum_{i=2}^{N}\frac{V_{i}(t)}{\left\|V_{i}(t)\right\|}\right\|,\;with\;V_{i}(t)=\left[\begin{array}{c} v_{xi}(t)\\ v_{yi}(t) \end{array}\right],$$

where the components of the vectors are explicitly indicated, *N* = 5, and the treated subject is assigned the index *i* = 1. The instantaneous polarization ranges from 0 to 1, where 1 indicates full alignment of swimming directions between the untreated fish and zero absence of alignment.

To study causal interactions within the group, we implemented a transfer entropy analysis following an ample body of literature on the subject (Pilkiewicz et al., 2020). In particular, transfer entropy quantifies the cause-and-effect relationship between two time series of length *N_d_*, ${\left\{s_{1}(k)\right\}}_{k=1}^{N_{d}}$ (effect) and ${\left\{s_{2}(k)\right\}}_{k=1}^{N_{d}}$ (cause), and it is defined as the extent by which knowledge of the presence of the cause reduces uncertainty in the prediction of the future of the effect from its present. Because the computation of transfer entropy entails the use of probabilities, we represent the time series *s*_1_(*k*) and *s*_2_(*k*) as two stationary stochastic processes *S*_1_(*k*) and *S*_2_(*k*), respectively (for further details, see Bossomaier et al., 2016). Then, transfer entropy (measured in bits) from *S*_2_(*k*) to *S*_1_(*k*) is given by

$$TE_{S_{2}\to S_{1}}=\sum_{S_{1}(k+1),S_{1}(k),S_{2}(k)}p\left(S_{1}(k+1),S_{1}(k),S_{2}(k)\right)\times \log_{2}\frac{p\left(S_{1}(k+1)|S_{1}(k),S_{2}(k)\right)}{p\left(S_{1}(k+1)|S_{1}(k)\right)}.$$

Here, *p* (*S*_1_ (*k* + 1 ), | *S*_1_ (*k*),*S*_2_ (*k*)) is a joint probability indicating how likely the realizations of these three variables *S*_1_(*k*+1), *S*_1_(*k*), and *S*_2_(*k*) occur. *p* (*S*_1_ (*k* + 1) | *S*_1_ (*k*), *S*_2_ (*k*)) is a conditional probability indicating how likely future realizations of *S_1_* (at time (*k*+1)) happen, given a particular value for the present realizations of *S_1_* and *S_2_* (at time *k*). Similarly, *p* (*S*_1_ (*k* + 1 | *S*_1_(k)) denotes the likelihood of future realizations of *S_1_*, given its present value at time *k*. Transfer entropy *TE_S_2_→S_1__* is zero if *S*_2_(*k*) does not cause *S*_1_(*k*), while a nonzero value of *TE_S_2_→S_1__* indicates the existence of a cause-and-effect relationship.

We computed transfer entropy among all fish in condition Groups for all the three Citalopram treatments; control, 30 (mg/L) and 100 (mg/L). Given our interest in detailing emotional contagion related to diving and rising patterns, we used the time series of velocity along the water column to compute transfer entropy. Based on our previous work (Porfiri and Marín, 2017) and the need to ensure Markovianity of the stochastic processes, we downsampled the time series of the velocity using a resolution of six time steps (time period of 0.2 s or 5 frames/s) yielding a total of *N_d_* = 3,000; this time period was informed by our previous studies (Butail et al., 2016; Mwaffo et al., 2017). To compute transfer entropy, we first binned the time series of the velocity of each individual using two bins, that is, ${\tilde{v}}_{yi}(k)$ = “−” if *v_yi_(k)* < 0, corresponding to motion towards the bottom of the tank and ${\tilde{v}}_{yi}(k)$ = “+” if *v_yi_(k)* ≥ 0, corresponding to motion towards the top. Using the time series of ${\tilde{v}}_{yi}(k)$ of each fish in the group, we calculated 20 values of transfer entropy aggregated in a five by five matrix *TE*. The diagonal elements of the matrix are zero, while the generic element off the diagonal, *TE_ij_*, corresponds to transfer entropy from individual *i* to individual *j*.

Ultimately, to quantify the overall influence of fish *i* on these of the group, we computed net transfer entropy for each individual (Mwaffo et al., 2017)

$$NetTE_{i}=\sum_{j=1,j\neq i}^{N}TE_{ij}-TE_{ji}.$$

For each trial, we computed five values of net transfer entropy, one for the treated subject and four for the untreated shoal members. We averaged the latter four values so that each trial would yield two measures of net transfer entropy quantifying the role of Citalopram administration on the influence of any focal subject (be it treated or not) on the other shoal members.

### Statistical Analyses

To investigate the role of the social context on individual response to Citalopram administration, we analyzed the distance from the bottom in fish in the condition Singles and in Citalopram-treated fish in the condition Groups. We conducted an analysis of the distance from the bottom using a two-way ANOVA with Citalopram concentration and social context as independent variables. To address the extent to which an individual exposed to Citalopram altered group behavior in terms of geotaxis, we compared the distance from the bottom of all the animals in condition Groups as a function of exposure to Citalopram. To account for the possibility that the group average was influenced by the treated subject, we also investigated the distance from the bottom response of the untreated subjects in the condition Groups. For these analyses, we utilized a one-way ANOVA with Citalopram concentration as the independent variable. Similarly, we used a one-way ANOVA to analyze variables associated with group cohesion and coordination; namely, the nearest neighbor distance to the treated subject, the nearest neighbor distance between untreated subjects, and polarization. Outlier values were identified as those above or below two standard deviations from the median of the distance from the bottom for condition Singles and Groups at each Citalopram concentration. One outlier was identified for condition Groups at 0 mg/L and discarded from the analysis. Net transfer entropy was analyzed using a two-way ANOVA where Citalopram concentration (0, 30, and 100 mg/L) and treatment administration (treated fish vs. untreated shoal members) were selected as independent variables. *Post hoc* comparisons were performed using the Tukey test.

Comparisons of nearest neighbor distances and polarization with chance were conducted using one-tailed *t*-tests with Holm corrections (Holm, 1979). To establish chance values, we follow these steps: (i) randomly choose five individuals among all 216 fish used in the experiment; (ii) calculate the average values of the nearest neighbor distance to a single fish, the nearest neighbor distance between four fish, and polarization; (iii) repeat steps (i)-(ii) 10,000 times; and (iv) calculate the average values of the 10,000 iterations. Net transfer entropy values were compared with chance using a two-tailed *t*-test. All statistical analyses were performed with the statistics software R (version 3.6.1) with a significance level of 0.050. Specifically, we used the following functions in R; *aov* for conducting all ANOVAs, *emmeans* with Tukey adjustment was used for *post hoc* analyses, and finally, the *t*-tests were conducted using *t*_test.

## Results

### Individual Response to Citalopram Varies as a Function of the Social Context

**Figure 2:**
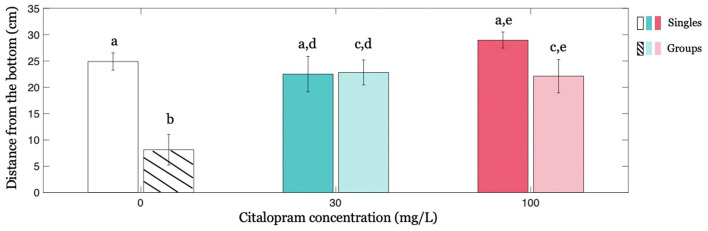
Individual geotaxis as a function of Citalopram treatment and social context. Distance from the bottom averaged across time as a function of Citalopram treatment in Singles and in focal subjects in condition Groups. Different letters indicate differences at *p* < 0.050.

When administered to zebrafish swimming in isolation (condition Singles), Citalopram did not affect the distance from the bottom of the tank. In particular, we did not register a statistically significant difference between Citalopram treatments (*F*_(2,32)_ = 1.882; *p* = 0.169; Figure 2).

Interestingly, we found that the social context, that is, the presence of four (untreated) subjects, altered the response of the focal individual. Specifically, while Citalopram-treated subjects and control individuals exhibited an indistinguishable distance from the bottom in condition Singles, Citalopram-treated subjects showed a greater distance from the bottom of the tank compared to vehicle-treated individuals in the condition Groups (Figure 2). Thus, for the focal fish in condition Groups, we registered a significantly different distance from the bottom between 0 mg/L and 30 mg/L (*p* < 0.050) and between 0 mg/L and 100 mg/L (*p* < 0.050). Additionally, when we compared the effect of the social context on the behavior of the treated subject, we observed that swimming alone or in a group remarkably altered the average distance from the bottom of vehicle-treated individuals but not of Citalopram-treated ones as indicated by the significant social context x Citalopram administration interaction (*F*_(2,64)_ = 5.535; *p* < 0.050) and further confirmed by *post hoc* analyses. Specifically, *post hoc* analyses revealed that vehicle-treated subjects swam closer to the bottom when they were in groups rather than alone (*p* < 0.001; Figure 2). This effect however was absent for 30 mg/L (*p* = 0.931) indicating that the presence of a shoal did not alter the behavior of the focal fish when treated with Citalopram for this concentration. For the higher Citalopram dose of 100 mg/L, we registered a trend (*p* = 0.065).

### Collective Geotactic Response Is Modulated by Citalopram Administration

**Figure 3:**
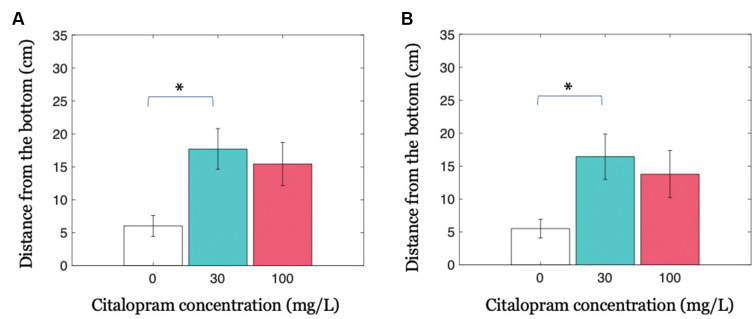
Group geotaxis as a function of Citalopram administration. **(A)** Distance from the bottom of the tank for condition Groups. **(B)** Distance from the bottom of the tank for the untreated members in condition Groups. *p < 0.050 compared to vehicle-treated subjects.

Our results indicate that the presence of a treated fish in the shoal decreases the anxiety-related behavior of the entire group in the form of increased distance from the bottom of the tank. When investigating this parameter in all shoal members, we observed that the intermediate Citalopram concentration (30 mg/L) resulted in reduced geotaxis compared to vehicle (Citalopram treatment: *F*_(2,32)_ = 4.702; *p* <0.050; *p* <0.050 in *post hoc* tests; Figure 3A). While experimental subjects exposed to 30 mg/L and 100 mg/L did not differ (*p* = 0.832), we observed a trend towards increased distance from the bottom of the tank 100 mg/L compared to 0 mg/L (*p* = 0.064).

Previous data may be partly explained by the possibility that the treated individual skewed the overall distribution. To account for this potential confound, we conducted an analysis only on the untreated shoal members in the three experimental groups. In accordance with the hypothesis that untreated subjects were influenced by the treated individual, we observed that untreated fish swimming together with a 30 mg/L Citalopram-treated individual displayed a higher distance from the bottom compared to untreated shoal members swimming with a vehicle-treated subject (Citalopram treatment: *F*_(2,32)_ = 3.384; *p* <0.050; *p* <0.050 in *post hoc* tests; Figure 3B). We did not register an equivalent effect in untreated fish swimming with a 100 mg/L Citalopram-treated individual (*p* = 0.155).

### Group Cohesion and Coordination Are Not Modulated by Citalopram Administration

**Figure 4:**
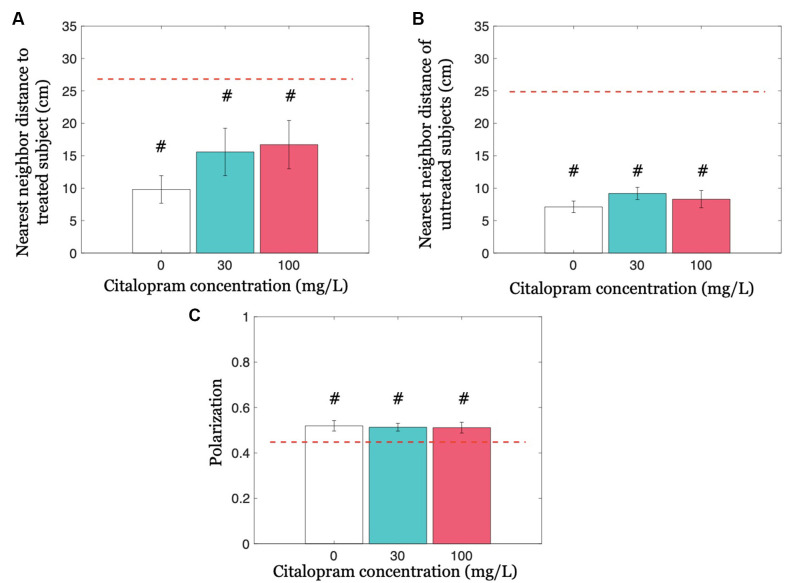
Group cohesion and coordination quantified as nearest neighbor distances and polarization, respectively. **(A)** The nearest neighbor distance to the treated subject for each Citalopram concentration. **(B)** Nearest neighbor distance of untreated subjects for each Citalopram concentration. **(C)** Polarization as a function of Citalopram concentration. #*p* < 0.050 compared to the chance value, denoted by the red dashed-line.

We first addressed whether Citalopram administration altered cohesion by analyzing the nearest neighbor distance to the treated fish, and afterward the nearest neighbor distance only between untreated subjects. Neither did Citalopram treatment modulate the nearest neighbor distance to the treated subject (*F*_(2,32)_ = 1.214; *p* = 0.310), nor did it alter the nearest neighbor distance of untreated subjects (*F*_(2,32)_ = 0.905; *p* = 0.415; Figures 4A,B). Similarly, experimental groups did not differ in terms of polarization (*F*_(2,32)_ = 0.037; *p* = 0.963; see Figure 4C).

We further compared cohesion and coordination variables with chance values. The neighbor distance to the treated subject was significantly lower than chance, for all three Citalopram treatments 0 mg/L (*t*_(10)_ = −8.030; *p* < 0.050), 30 mg/L (*t*_(11)_ = −3.060; *p* < 0.050), and 100 mg/L (*t*_(11)_ = −2.720; *p* < 0.050). We also observed a significant difference from chance with respect to the nearest neighbor distance of untreated subjects at each Citalopram concentration (*p* < 0.050). Likewise, we found that polarization differed from the chance for each Citalopram concentration (*p* < 0.050).

### Causal Interactions Within the Group as a Function of Citalopram Administration

**Figure 5:**
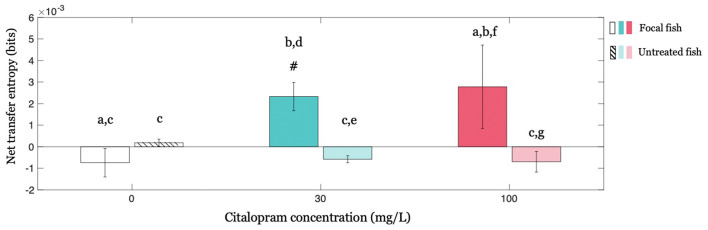
Causal interactions within the group revealed by net transfer entropy between the focal fish and all other animals in the group for each Citalopram concentration. Positive values of net transfer entropy indicate the influence of the focal subject (treated or untreated) on the rest of the group while negative values indicate the opposite, that is, the focal subject being influenced by the others. #*p* < 0.050 greater than zero, and different letters indicate differences at *p* < 0.050.

We investigated whether the treated (focal) individual influenced untreated shoal members by studying the corresponding net transfer entropies and investigating the potential modulatory role of Citalopram. Our results show that the influence exerted by the Citalopram-treated fish on the group of untreated fish varied as a function of Citalopram concentration (interaction between Citalopram concentration (0, 30, and 100 mg/L) and treatment administration (treated vs. untreated; *F*_(2,64)_ = 3.284; *p* < 0.050) in net transfer entropy). The main effect of treatment administration was significant (*F*_(1,64)_ = 6.368; *p* < 0.050), while the main effect of Citalopram concentration did not attain statistical significance (*F*_(2,64)_ = 1.182; *p* = 0.313). Conducting *post hoc* analyses, we observed that net transfer entropy from the Citalopram-treated subjects to the untreated members was higher than net transfer entropy from untreated to treated for Citalopram concentrations of 30 mg/L (*p* < 0.050) and 100 mg/L (*p* < 0.050); this difference was absent in vehicle-treated fish (*p* = 0.492; Figure 5). Additionally, while untreated subjects displayed an equivalent net transfer entropy across Citalopram concentrations (0, 30, and 100 mg/L), focal fish treated with Citalopram displayed higher net transfer entropy compared to vehicle-treated individuals. Thus, we observed a different net transfer entropy between 0 mg/L and 30 mg/L (*p* < 0.050), along with a trend in the comparison between 0 mg/L and 100 mg/L (*p* = 0.058). Ultimately, we found that net transfer entropy is significantly higher than zero for the treated fish at 30 mg/L (*t*_(11)_ = 3.560; *p* < 0.050), but not for vehicle (*t*_(10)_ = −1.130; *p* = 0.857) and 100 mg/L (*t*_(11)_ = 1.440; *p* = 0.088).

## Discussion

Emotional contagion, the unconscious/conscious process by which we adjust our behavior in accordance with that exhibited by others, is one of the building blocks of social behavior (Hatfield et al., 2011). Emotional contagion can influence social decision making and overall group performance through the spread of positive or negative emotions, which impact morale, motivation, and rapport. While this phenomenon has been widely documented across several taxa such as mammals (Langford et al., 2006; Zoratto et al., 2018) and birds (Adriaense et al., 2019), limited evidence of its occurrence has been reported in fish (Pérez-Manrique and Gomila, 2021). In an effort to contribute to a better understanding of emotional contagion, we studied how anxiety-related behavior can be propagated in groups of zebrafish by pharmacologically manipulating the behavior of a focal individual in the shoal. Zebrafish is a highly social species which possesses a complex behavioral repertoire making it a suitable option to study emotional contagion.

Our main aim was to induce an overt phenotype in a single individual and evaluate the extent to which this manipulation translated into a behavioral change in a group of naïve individuals. Specifically, we hypothesized that a fish exhibiting low levels of anxiety would influence naïve individuals such that the latter would also exhibit low anxiety states as a proxy for emotional contagion. To achieve this aim, we administered Citalopram to a single fish swimming in a group of untreated subjects and examined the geotactic response (the distance from the bottom of the tank) both in terms of absolute values and net transfer entropy. Geotaxis is considered a very sensitive behavioral indicator of anxiety-related states (Kalueff et al., 2013). It has been validated in a wide range of experimental paradigms (Clèment et al., 2020; Karakaya et al., 2021), and it has been repeatedly reported to vary as a function of the administration of selective serotonin reuptake inhibitors like Citalopram (Wong et al., 2010; Cachat et al., 2011; Kalueff et al., 2016; Collier et al., 2017; Macrì et al., 2019). To further delve into the nature of shoaling and schooling tendencies, we conducted a thorough analysis of polarization (Aureli et al., 2012) and neighbor distances (Rosa et al., 2020).

In accordance with our predictions, we observed that a Citalopram-treated fish that exhibits low levels of anxiety-related behaviors apparently transfers a similar phenotype to a group of untreated individuals. In particular, while we observed that untreated subjects swimming together with a vehicle-treated individual exhibit a strong geotactic activity throughout the entire experimental session, untreated subjects swimming with a Citalopram-treated fish, spend more time in the upper part of the experimental tank. While attempting to describe the nature of treated-to-untreated information spreading, we first focused on the treated subjects and then on parameters associated with shoaling and schooling tendencies. Preliminarily, we observed that while vehicle-treated individuals were influenced by their group whereby they swam closer to the bottom of the tank compared to when they were swimming alone, Citalopram treated individuals were insensitive to the group.

This indication of the influential role played by Citalopram-treated subjects was further strengthened by the net transfer entropy analysis. Transfer entropy is an information-theoretic concept that is used to infer directional interactions between variables in the form of causal influence. Specifically, transfer entropy measures the extent to which knowledge of the present state of a causal variable helps reduce the uncertainty associated with the prediction of the future of an effect variable from its own state (Bossomaier et al., 2016). Transfer entropy has been employed across a wide range of animal behavior studies to dissect the modality of interactions between two or multiple individuals (Pilkiewicz et al., 2020). We pursued the transfer entropy analysis on symbolic time-series associated with diving and rising patterns to study geotaxis within the context of emotional contagion. The transfer entropy analysis supports the emergence of a directional influence of the treated individual on the group and its dependence on Citalopram concentration. Although the treated individual swims in the same tank as the untreated subjects, it virtually acts as a demonstrator for them, whereby it influenced their behavior but not vice-versa. Such an influence would be masked had we analyzed only distances and polarization, whereby these variables were not modulated by Citalopram administration. This evidence calls once again for multi-target tracking of individual response for the study of social behavior (Hughey et al., 2018; Franco-Restrepo et al., 2019) and the value of data-driven, model-free techniques to quantify social interactions (Strandburg-Peshkin et al., 2018; Pilkiewicz et al., 2020).

Although emotional contagion was initially believed to be a prerogative of mammals and birds, recent empirical evidence demonstrates that simpler animals (Rose, 2002; Rose et al., 2014), are able to share emotions as well (Oliveira et al., 2017; Silva et al., 2019; Pérez-Manrique and Gomila, 2021). Our study complements these efforts and provides evidence of emotional contagion in the form of a reduction of anxiety-related behaviors propagating from a single, Citalopram-treated zebrafish to a group of untreated conspecifics. These findings parallel earlier evidence collected in mice. For instance, Langford et al. (2006) showed that a single mouse treated with a painful agent (acetic acid) experiences reduced pain in the presence of a control mouse than when tested in isolation. The painful response was instead amplified if the treated mouse was in visual contact with a conspecific exposed to the same treatment. As pointed out by Nakahashi and Ohtsuki (2015), this behavior is believed to operate unconsciously and rest upon the amygdala, which is known to play a key role in emotional learning and social behavior (Perathoner et al., 2016). Since Citalopram acts on the amygdala in mammals (Murphy et al., 2009) and since zebrafish possess a homolog of the amygdala [a subpopulation of neurons in the medial zone of the dorsal telencephalon (Lal et al., 2018)], it is tenable that the phenotypes observed in our study are isomorphic to those observed in other species.

The experimental setup that we devised to study emotional contagion consisted of a thin water tank to promote motion along the water column against cross-section sweeping. We cannot exclude the possibility that constraining the motion of the group on a nearly 2D plane might have promoted emotional contagion against other forms of collective behavior. Future studies should investigate analogous phenotypes in experimental tanks allowing the exhibition of a complete 3D behavioral repertoire. The analysis of such experiments shall employ dedicated 3D tracking systems, which have been shown to unravel behavioral patterns hidden by 2D analysis (Macrì et al., 2017, 2019; Rosa et al., 2020). Additionally, our study leveraged a pharmacological manipulation to maximize the exhibition of a desired phenotype. Similar to Oliveira et al. (2017), future studies shall address the extent to which this form of emotional contagion extends to phenotypes induced by natural stimuli, like the view or odor of a predator. Despite these avenues of potential inquiry, this effort offers an empirical basis to study emotional contagion in zebrafish, beyond the demonstrator/bystander dichotomy and fear contagion-based approaches that form the large majority of existing literature.

## Data Availability Statement

The datasets presented in this study can be found in the online repository: https://github.com/dynamicalsystemslaboratory/EmotionalContagionZebrafish.

## Ethics Statement

The animal study was reviewed and approved by the University Animal Welfare Committee (UAWC) of New York University under protocol number 13-1424. All experiments were conducted at New York University, Tandon School of Engineering in Brooklyn, NY, USA.

## Author Contributions

All the authors contributed to the article. SM and MP designed the research. All the authors formulated the hypotheses of the study. DABL developed the experimental setup, conducted the experiments, conducted statistical analysis, and prepared a first, preliminary draft of the manuscript. SM and MP consolidated the first draft into the final submission. All the authors analyzed the data, discussed the results, and reviewed and approved the submitted version.

## Conflict of Interest

The authors declare that the research was conducted in the absence of any commercial or financial relationships that could be construed as a potential conflict of interest.

## Publisher’s Note

All claims expressed in this article are solely those of the authors and do not necessarily represent those of their affiliated organizations, or those of the publisher, the editors and the reviewers. Any product that may be evaluated in this article, or claim that may be made by its manufacturer, is not guaranteed or endorsed by the publisher.

## References

[B1] Adriaense J. E. Martin J. S. Schiestl M. Lamm C. Bugnyar T. (2019). Negative emotional contagion and cognitive bias in common ravens (Corvus corax). Proc. Natl. Acad. Sci. U S A 116, 11547–11552. 10.1073/pnas.1817066116 31110007 PMC6561263

[B2] Aleström P. D’angelo L. Midtlyng P. J. Schorderet D. F. Schulte-Merker S. Sohm F.. (2020). Zebrafish: housing and husbandry recommendations. Lab. Anim. 54, 213–224. 10.1177/0023677219869037 31510859 PMC7301644

[B3] Aureli M. Fiorilli F. Porfiri M. (2012). Portraits of self-organization in fish schools interacting with robots. Physica D 241, 908–920. 10.1016/j.physd.2012.02.005

[B4] Avdesh A. Chen M. Martin-Iverson M. T. Mondal A. Ong D. Rainey-Smith S.. (2012). Regular care and maintenance of a zebrafish (*Danio rerio*) laboratory: an introduction. J. Vis. Exp. e4196. 10.3791/4196 23183629 PMC3916945

[B5] Bakker A. B. Schaufeli W. B. (2000). Burnout contagion processes among teachers. J. Appl. Soc. Psychol. 30, 2289–2308. 10.1111/j.1559-1816.2000.tb02437.x

[B6] Barsade S. G. (2002). The ripple effect: emotional contagion and its influence on group behavior. Admin. Sci. Quart. 47, 644–675. 10.2307/3094912

[B7] Bartolini T. Butail S. Porfiri M. (2015). Temperature influences sociality and activity of freshwater fish. Env. Biol. Fishes 98, 825–832. 10.1007/s10641-014-0318-8

[B8] Bossomaier T. Barnett L. Harré M. Lizier J. T. (2016). An Introduction to Transfer Entropy. Switzerland: Springer.

[B9] Brothers L. (1989). A biological perspective on empathy. Am. J. Psychiatry 146, 10–19. 10.1176/ajp.146.1.10 2643353

[B10] Butail S. Bartolini T. Porfiri M. (2013). Collective response of zebrafish shoals to a free-swimming robotic fish. PLoS One 8:e76123. 10.1371/journal.pone.0076123 24146825 PMC3797741

[B11] Butail S. Mwaffo V. Porfiri M. (2016). Model-free information-theoretic approach to infer leadership in pairs of zebrafish. Phys. Rev. E 93:042411. 10.1103/PhysRevE.93.042411 27176333

[B12] Cachat J. M. Canavello P. R. Elkhayat S. I. Bartels B. K. Hart P. C. Elegante M. F.. (2011). “Video-aided analysis of zebrafish locomotion and anxiety-related behavioral responses,” in Zebrafish Neurobehavioral Protocols, (Totowa, NJ: Humana Press), 1–14.

[B13] Choi T.-Y. Choi T.-I. Lee Y.-R. Choe S.-K. Kim C.-H. (2021). Zebrafish as an animal model for biomedical research. Exp. Mol. Med. 53, 310–317. 10.1038/s12276-021-00571-5 33649498 PMC8080808

[B14] Clèment R.J. Macrì S. Porfiri M. (2020). Design and development of a robotic predator as a stimulus in conditioned place aversion for the study of the effect of ethanol and citalopram in zebrafish. Behav. Brain Res. 378:112256. 10.1016/j.bbr.2019.112256 31614187 PMC6893136

[B15] Collier A. D. Kalueff A. V. Echevarria D. J. (2017). “Zebrafish models of anxiety-like behaviors,” in The Rights and Wrongs of Zebrafish: Behavioral Phenotyping of Zebrafish, (Switzerland: Springer), 45–72.

[B16] Decety J. Ickes W. (2011). The Social Neuroscience of Empathy. Cambridge, MA: MIT Press.

[B17] Ferrari P. F. Gallese V. Rizzolatti G. Fogassi L. (2003). Mirror neurons responding to the observation of ingestive and communicative mouth actions in the monkey ventral premotor cortex. Eur. J. Neurosci. 17, 1703–1714. 10.1046/j.1460-9568.2003.02601.x 12752388

[B18] Fontana B. D. Mezzomo N. J. Kalueff A. V. Rosemberg D. B. (2018). The developing utility of zebrafish models of neurological and neuropsychiatric disorders: A critical review. Exp. Neurol. 299, 157–171. 10.1016/j.expneurol.2017.10.004 28987462

[B19] Franco-Restrepo J. E. Forero D. A. Vargas R. A. (2019). A review of freely available, open-source software for the automated analysis of the behavior of adult zebrafish. Zebrafish 16, 223–232. 10.1089/zeb.2018.1662 30625048

[B20] Gonzalez-Liencres C. Juckel G. Tas C. Friebe A. Brüne M. (2014). Emotional contagion in mice: the role of familiarity. Behav. Brain Res. 263, 16–21. 10.1016/j.bbr.2014.01.020 24480421

[B21] Hatfield E. Cacioppo J. T. Rapson R. L. (1994). Emotional Contagion. Cambridge, United Kingdom: Cambridge University Press.

[B22] Hatfield E. Rapson R. L. Le Y.-C. L. (2011). “Emotional contagion and empathy,” in The Social Neuroscience of Empathy, eds Decety J. Ickes W. (Cambridge, MA: MIT press), 19–30.

[B23] Holm S. (1979). A simple sequentially rejective multiple test procedure. Scand. J. Stat. 6, 65–70.

[B24] Howe K. Clark M. D. Torroja C. F. Torrance J. Berthelot C. Muffato M.. (2013). The zebrafish reference genome sequence and its relationship to the human genome. Nature 496, 498–503. 10.1038/nature12111 23594743 PMC3703927

[B25] Hughey L. F. Hein A. M. Strandburg-Peshkin A. Jensen F. H. (2018). Challenges and solutions for studying collective animal behaviour in the wild. Philos. Trans. R Soc. Lond. B Biol. Sci. 373:20170005. 10.1098/rstb.2017.0005 29581390 PMC5882975

[B26] Kalueff A. V. Echevarria D. J. Homechaudhuri S. Stewart A. M. Collier A. D. Kaluyeva A. A.. (2016). Zebrafish neurobehavioral phenomics for aquatic neuropharmacology and toxicology research. Aquat. Toxicol. 170, 297–309. 10.1016/j.aquatox.2015.08.007 26372090

[B27] Kalueff A. V. Gebhardt M. Stewart A. M. Cachat J. M. Brimmer M. Chawla J. S.. (2013). Towards a comprehensive catalog of zebrafish behavior 1.0 and beyond. Zebrafish 10, 70–86. 10.1089/zeb.2012.0861 23590400 PMC3629777

[B28] Karakaya M. Scaramuzzi A. Macrì S. Porfiri M. (2021). Acute Citalopram administration modulates anxiety in response to the context associated with a robotic stimulus in zebrafish. Prog. Neuropsychopharmacol. Biol. Psychiatry 108:110172. 10.1016/j.pnpbp.2020.110172 33188831 PMC8026524

[B29] Lal P. Tanabe H. Suster M. L. Ailani D. Kotani Y. Muto A.. (2018). Identification of a neuronal population in the telencephalon essential for fear conditioning in zebrafish. BMC Biol. 16:45. 10.1186/s12915-018-0502-y 29690872 PMC5978991

[B30] Langford D. J. Crager S. E. Shehzad Z. Smith S. B. Sotocinal S. G. Levenstadt J. S.. (2006). Social modulation of pain as evidence for empathy in mice. Science 312, 1967–1970. 10.1126/science.1128322 16809545

[B31] Macrì S. Clèment R. J. Spinello C. Porfiri M. (2019). Comparison between two-and three-dimensional scoring of zebrafish response to psychoactive drugs: identifying when three-dimensional analysis is needed. PeerJ. 7:e7893. 10.7717/peerj.7893 31637136 PMC6800527

[B32] Macrì S. Neri D. Ruberto T. Mwaffo V. Butail S. Porfiri M. (2017). Three-dimensional scoring of zebrafish behavior unveils biological phenomena hidden by two-dimensional analyses. Sci. Rep. 7:1962. 10.1038/s41598-017-01990-z 28512334 PMC5434067

[B33] Mathuru A. S. Schirmer A. Tabitha N. P. Y. Kibat C. Cheng R.-K. Jesuthasan S. (2017). Familiarity with companions aids recovery from fear in zebrafish. bioRxiv [Preprint]. 10.1101/098509

[B34] Miller N. Gerlai R. (2012). From schooling to shoaling: patterns of collective motion in zebrafish (*Danio rerio*). PLoS One 7:e48865. 10.1371/journal.pone.0048865 23166599 PMC3498229

[B35] Miller R. E. Banks J. H. Jr Ogawa N. (1963). Role of facial expression in "cooperative-avoidance conditioning" in monkeys. J. Abnorm. Soc. Psychol. 67, 24–30. 10.1037/h0044018

[B36] Mirsky I. A. Miller R. E. Murphy J. V. (1958). The communication of affect in rhesus monkeys: I. an experimental method. J. Am. Psychoanal. Assoc. 6, 433–441. 10.1177/000306515800600303 13575267

[B37] Mogil J. S. (2012). The surprising empathic abilities of rodents. Trends Cogn. Sci. 16, 143–144. 10.1016/j.tics.2011.12.012 22206750

[B38] Mogil J. S. (2019). Mice are people too: increasing evidence for cognitive, emotional and social capabilities in laboratory rodents. Canad. Psychol./Psychologie Canadienne 60, 14–20. 10.1037/cap0000166

[B39] Murphy S. E. Norbury R. O’sullivan U. Cowen P. J. Harmer C. J. (2009). Effect of a single dose of citalopram on amygdala response to emotional faces. Br. J. Psychiatry 194, 535–540. 10.1192/bjp.bp.108.056093 19478294 PMC2802527

[B40] Mwaffo V. Butail S. Porfiri M. (2017). Analysis of pairwise interactions in a maximum likelihood sense to identify leaders in a group. Front. Robot. AI 4:35. 10.3389/frobt.2017.00035

[B41] Nakahashi W. Ohtsuki H. (2015). When is emotional contagion adaptive. J. Theor. Biol. 380, 480–488. 10.1016/j.jtbi.2015.06.014 26113192

[B42] Norton W. Bally-Cuif L. (2010). Adult zebrafish as a model organism for behavioural genetics. BMC Neurosci. 11:90. 10.1186/1471-2202-11-90 20678210 PMC2919542

[B43] Nusslein-Volhard C. Dahm R. (2002). Zebrafish. New York, United States: Oxford University Press Inc.

[B44] Oliveira T. A. Idalencio R. Kalichak F. Dos Santos Rosa J. G. Koakoski G. De Abreu M. S.. (2017). Stress responses to conspecific visual cues of predation risk in zebrafish. PeerJ. 5:e3739. 10.7717/peerj.3739 28890851 PMC5588784

[B45] Palagi E. Celeghin A. Tamietto M. Winkielman P. Norscia I. (2020). The neuroethology of spontaneous mimicry and emotional contagion in human and non-human animals. Neurosci. Biobehav. Rev. 111, 149–165. 10.1016/j.neubiorev.2020.01.020 31972204

[B46] Palagi E. Nicotra V. Cordoni G. (2015). Rapid mimicry and emotional contagion in domestic dogs. R. Soc. Open Sci. 2:150505. 10.1098/rsos.150505 27019737 PMC4807458

[B47] Panula P. Chen Y.-C. Priyadarshini M. Kudo H. Semenova S. Sundvik M.. (2010). The comparative neuroanatomy and neurochemistry of zebrafish CNS systems of relevance to human neuropsychiatric diseases. Neurobiol. Dis. 40, 46–57. 10.1016/j.nbd.2010.05.010 20472064

[B48] Parrish J. K. Viscido S. V. Grunbaum D. (2002). Self-organized fish schools: an examination of emergent properties. Biol. Bull. 202, 296–305. 10.2307/1543482 12087003

[B49] Perathoner S. Cordero-Maldonado M. L. Crawford A. D. (2016). Potential of zebrafish as a model for exploring the role of the amygdala in emotional memory and motivational behavior. J. Neurosci. Res. 94, 445–462. 10.1002/jnr.23712 26833658

[B50] Pérez-Manrique A. Gomila A. (2021). Emotional contagion in nonhuman animals: a review. Wiley Interdiscip. Rev. Cogn. Sci. e1560. 10.1002/wcs.1560 33951303 PMC9285817

[B51] Pilkiewicz K. Lemasson B. Rowland M. Hein A. Sun J. Berdahl A.. (2020). Decoding collective communications using information theory tools. J. R. Soc. Interface 17:20190563. 10.1098/rsif.2019.0563 32183638 PMC7115225

[B52] Porfiri M. Marín M. R. (2017). Symbolic dynamics of animal interaction. J. Theor. Biol. 435, 145–156. 10.1016/j.jtbi.2017.09.005 28916452

[B53] Rosa L. V. Costa F. V. Canzian J. Borba J. V. Quadros V. A. Rosemberg D. B. (2020). Three-and bi-dimensional analyses of the shoaling behavior in zebrafish: influence of modulators of anxiety-like responses. Prog. Neuropsychopharmacol. Biol. Psychiatry 102:109957. 10.1016/j.pnpbp.2020.109957 32360787

[B54] Rose J. D. (2002). The neurobehavioral nature of fishes and the question of awareness and pain. Rev. Fisheries Sci. 10, 1–38. 10.1080/20026491051668

[B55] Rose J. D. Arlinghaus R. Cooke S. J. Diggles B. K. Sawynok W. Stevens E.. (2014). Can fish really feel pain. Fish Fisheries 15, 97–133. 10.1111/faf.12010

[B56] Sackerman J. Donegan J. J. Cunningham C. S. Nguyen N. N. Lawless K. Long A.. (2010). Zebrafish behavior in novel environments: effects of acute exposure to anxiolytic compounds and choice of *Danio rerio* line. Int. J. Comp. Psychol. 23:43. 20523756 PMC2879659

[B57] Silva P. F. De Leaniz C. G. Luchiari A. C. (2019). Fear contagion in zebrafish: a behaviour affected by familiarity. Anim. Behav. 153, 95–103. 10.1101/521187

[B58] Stewart A. M. Braubach O. Spitsbergen J. Gerlai R. Kalueff A. V. (2014). Zebrafish models for translational neuroscience research: from tank to bedside. Trends Neurosci. 37, 264–278. 10.1016/j.tins.2014.02.011 24726051 PMC4039217

[B59] Strandburg-Peshkin A. Papageorgiou D. Crofoot M. C. Farine D. R. (2018). Inferring influence and leadership in moving animal groups. Philos. Trans. R Soc. Lond B Biol. Sci. 373:20170006. 10.1098/rstb.2017.0006 29581391 PMC5882976

[B60] Theodorakis C. W. (1989). Size segregation and the effects of oddity on predation risk in minnow schools. Anim. Behav. 38, 496–502. 10.1016/s0003-3472(89)80042-9

[B61] Von Scheve C. Salmella M. (2014). Collective Emotions: Perspectives from Psychology, Philosophy and Sociology. New York, United States: Oxford University Press Inc.

[B62] Wong K. Elegante M. Bartels B. Elkhayat S. Tien D. Roy S.. (2010). Analyzing habituation responses to novelty in zebrafish (*Danio rerio*). Behav. Brain Res. 208, 450–457. 10.1016/j.bbr.2009.12.023 20035794

[B63] Zoratto F. Sbriccoli M. Martinelli A. Glennon J. C. Macrì S. Laviola G. (2018). Intranasal oxytocin administration promotes emotional contagion and reduces aggression in a mouse model of callousness. Neuropharmacology 143, 250–267. 10.1016/j.neuropharm.2018.09.010 30213592

